# The 3-min all-out test in cycling: a systematic review and meta-analysis on its reliability and validity

**DOI:** 10.1007/s00421-026-06356-w

**Published:** 2026-07-21

**Authors:** Oliver J. Quittmann, Moritz A. Piehl

**Affiliations:** 1 Institute of Movement and Neurosciences, German Sport University Cologne, Cologne, Germany; 2 Exercise Science, Coaching and Performance Enhancement (E.S.C.A.P.E.) network, Cologne, Germany

**Keywords:** Critical power, Anaerobic capacity, W’, Sprint test, Power output, Exercise testing, Power profile

## Abstract

The 3-min all-out test is considered a time-efficient alternative for estimating critical power (CP) and curvature constant (Wʹ), by means of endtest power (EP) and work done above endtest power (WEP), respectively. This review aims to assess its reliability and validity with respect to methodology. Following the PRISMA 2020 guidelines, a systematic literature search was conducted on June 20th 2025, using a specified search term in PubMed and Web of Science. Included studies were English-language, peer-reviewed original articles, that provided empirical data on the 3-min all-out test in cycling and used appropriate protocols for assessing reliability/validity. Quality of studies was assessed using a modified critical appraisal tool. Meta-analysis for validity was done using pooled Hedge’s g_z_ with random effects synthesis and 95%prediction intervals. Twenty studies comprising N = 284 participants met all inclusion criteria. Thereof, 15 studies examined validity, 8 reliability and 3 both. Included studies demonstrated methodological heterogeneity and small sample sizes (n = 6–21). Test-retest reliability demonstrated ‘good’ to ‘excellent’ agreement for EP (ICC = 0.89–0.99) and WEP (ICC = 0.76–0.98). EP systematically overestimates CP (g_z_= 0.408 [0.024, 0.792]) while WEP underestimates W’ (g_z_= –0.389 [–0.954, 0.176]). Whereas the 3-min all-out test provides reliable results for EP and WEP if tests are conducted within three weeks, its validity seems to largely depend on the methodological approach. Certain power profiles and/or isokinetic 3-min tests lead to better estimates of CP, while the variability of WEP does not allow to accurately estimate Wʹ. Familiarisation trials and proper verbal encouragement during the 3-min test are mandatory.

**Supplementary Information:**

The online version contains supplementary material available at https://doi.org/10.1007/s00421-026-06356-w.

## Introduction

In endurance sports, performance diagnostic is an important tool for athletes and coaches. Various models have been proposed to assist with this process by providing structured frameworks (Jones et al. 2019a; Joyner 1991; Drake et al. 2024). Most concepts require the measurement gas exchange and lactate concentration, are expensive and require complex laboratory testing. In contrast, the critical power (CP) concept provides an alternative that does not rely on physiological measurements (Jones and Vanhatalo 2017). Instead, it focusses on external performance output rather than internal physiological responses. In a landmark paper, Monod and Scherrer (1965) stated that CP could be sustained without fatigue, leading to the assumption that for all power outputs at or below CP, exhaustion cannot occur (Monod and Scherrer 1965). Therefore, CP demarcates the transition from the ‘heavy’ to the ‘severe’ exercise domain (Jones et al. 2019a). In contrast to CP, the curvature constant (Wʹ) is viewed as a finite (anaerobic) energy reservoir that terminates the time at a given work rate above CP (Clark et al. 2013). Accordingly, knowledge of CP and Wʹ allows for accurate predictions of power output and/or finishing times and thus supports the development of tactical, positional and pacing strategies in competition (Jones et al. 2010; Jones and Vanhatalo 2017).

Even though the CP-approach is appealing in its simple nature, there are still multiple (typically between three to five) exhausting trials to be performed (typically ranging from 2 to 15 min) (Dekerle et al. 2008). Since these trials may cause severe fatigue and potentially interference with the athletes’ training schedule, practitioners and researchers seek for exercise tests that minimise test duration and still yield in reliable, valid and meaningful outcomes. As a result, the 3-min all-out test was developed in 2006 to measure CP and Wʹ as well as peak oxygen uptake (V̇O_2peak_) within a single session (Burnley et al. 2006). Based on the power-duration relationship, the 3-min all-out is designed to fully deplete the anaerobic work capacity within three minutes (Vanhatalo et al. 2007). Consequently, the endtest power (EP) estimates CP and is calculated as the average power output over the final 30 s of the test (Vanhatalo et al. 2007). Further, the work performed above endtest power (WEP) is considered to be similar to Wʹ and is calculated as the power-time integral above EP (Vanhatalo et al. 2007).

Due to the shortened protocol compared with the original standard of determining CP and Wʹ (Hill 1993), the 3-min all-out has gained attention over the past 20 years. Despite its popularity, reliability and validity have yet to bet ensured in order to recommend the 3-min all-out test as a practical tool. Although several primary studies have investigated the 3-min all-out regarding reliability and validity, to date, no comprehensive review has synthesised these findings. In 2025, a narrative review was published regarding the application of the 3-min all-out with a focus on prescribing high-intensity interval training (Pettitt et al. 2025). Another narrative review was published for training prescription using CP and Wʹ, as well as their reconstitution rates (Chorley and Lamb 2020). However, regarding these quality criteria, the current literature presents inconsistent findings. While some studies support the test outcomes (Johnson et al. 2011; Clark et al. 2016) other studies question their accuracy (Bartram et al. 2017; Maunder et al. 2022). Thus, a structured synthesis is warranted to evaluate reliability and – especially – validity.

Therefore, the purpose of this systematic review and meta-analysis is to assess the scientific literature concerning the reliability and validity of the 3-min all-out test in cycling. Given the methodological differences and inconsistent findings in the current literature (Clark et al. 2013; Vanhatalo et al. 2007; Johnson et al. 2011; Bartram et al. 2017; Maunder et al. 2022), it also aims to examine the effect of methodological approaches on the test outcomes and provide guidelines for appropriate testing setups. Although the 3-min all-out allows for the determination of multiple parameters, this systematic review focusses on the determination of EP and WEP. Concerning determination of peak power (P_max_) and maximal oxygen uptake (V̇O_2max_) an additional section is provided within the discussion.

## Methods

To improve reporting transparency, this systematic review followed the updated 2020 guidelines of the Preferred Reporting Items for Systematic Reviews and Meta-Analyses (PRISMA) statement (Page et al. 2021).

### Eligibility criteria

Inclusion criteria were defined as follows: (1) peer-reviewed articles; (2) original research articles (no review articles); (3) English language; (4) collecting empirical data of the 3-min all-out test in cycling; (5) appropriate test protocol for assessing reliability/validity; (6) appropriate reference test; and (7) providing data on reliability and/or validity. Accordingly, studies that did not meet the predefined inclusion criteria were excluded. Subsequently, the studies found were categorised into those regarding reliability, those examining validity and those assessing both.

### Information sources

The article search was conducted on June 20th, 2025, using PubMed and Web of Science.

### Search strategy

To systematically identify studies relevant to the research question, the following specific search string was applied to both databases: (“3-Minute All Out” OR “3-min all-out” OR “three-minute all-out” OR “all-out 3-min” OR “3MT " OR “3AOT” OR “AO3”) AND (“cycl*”) AND (“valid*” OR “reliab*”). Additionally, the filter was set to only find results from 2006 onward, as this was the year the test was introduced (Burnley et al. 2006).

### Selection process

The identified studies were imported into a reference management file (Zotero, 7.0.16, Digital Scholar, Vienna, Virginia, USA) and duplicates were removed manually. No automation tools were used. Studies were screened based on title and abstract. In the screening process, the authors excluded all articles with clear irrelevance to this research topic. Subsequently, full texts were sought for retrieval. The eligibility assessment was supported by a color-coding system to differentiate between included and (pre-)excluded studies. Again, no automation tools were used. The rationale for excluded articles was documented.

### Data collection process

To visualise similarities and differences, relevant data for comparing validity or reliability was extracted from full texts and tables. The data was transferred into customised tables using EXCEL (version 16.98, Microsoft, Redmond, WA, USA), one regarding reliability and one validity, respectively.

### Data items

Both tables contained the same categories: the sample size and the portion of female participants within; the performance level (PL) of the sample; the type of ergometer used for testing; the test mode (linear, isokinetic or freely geared); the measured outcome variables; and the reported statistical values. Additionally, the reliability table contained a column regarding the inter-test duration while the validity table included the criterion(s) used for comparison with the outcome variables.

PLs were categorised according to the guidelines to classify male subjects (De Pauw et al. 2013a) and female subjects (Decroix et al. 2016). Based on physiological performance indicators like V̇O_2max_ or cycling status like hours/distance per week participants were allocated between PL 1 (sedentary) and PL 5 (elite). If the indicators suggested different PLs, average relative V̇O_2max_ was used as the primary indicator. If only absolute V̇O_2max_ and body weight were reported, relative V̇O_2max_ was calculated. If values for male and female participants were not reported separately, the classification table for males was used, unless >50% of the participants were female.

### Study risk of bias assessment

To assess the methodological quality of the included literature, a risk of bias analyses was conducted. Therefore, the included articles were independently assessed by two raters following a modified version of the critical appraisal tool (Brink and Louw 2012). The tool contains 13 quality criteria and is suitable for studies focusing on parameters of reliability and validity. In case of inter-rater disagreement, a consensus was found through discussion. For the assessment, a customised EXCEL sheet was used. The independent ratings were based on 9 out of the 13 items: 1) description of sample; 3) explanation of reference standard; 7) short time period between tests; 8) stability of variable being between repeated measures; 9) reference standard independent of index test; 10) test execution described in detail; 11) reference standard described in detail; 12) withdrawals explained; 13) statistical methods appropriate for purpose. Items 2 (competence of raters), 4 (blinding of raters to findings of other raters), 5 (raters blinded to their own prior findings) and 6 (order of examination varied) were not assessed due to a lack of applicability to the studies included in this review. Items 1, 8, 10, 12 and 13 were used to assess reliability studies and items 1, 3, 7, 9, 10, 11, 12 and 13 were used to assess validity studies.

### Effect measures

The reliability table presents the outcomes of the studies primarily using intraclass correlation coefficients (ICCs). ICCs were interpreted according to Koo and Li (2016): ICC <0.5 = 'poor', 0.5–0.75 = 'moderate', 0.75–0.90 = 'good', and >0.90 = 'excellent' reliability (Koo and Li 2016). If ICCs were not reported, correlation coefficients (r) are provided in the column. The r-values were interpreted following Cohen (1988) with ≥0.1, ≥0.3 and ≥0.5 providing small, moderate and strong correlations, respectively (Cohen 1988).

For summarising the agreement between 3-min all-out parameters and other physiological characteristics, both limits of agreement (LoA) and determination coefficients (R²) are shown. LoA are presented with bias and either standard deviation or ranges. R²-values are presented in %. Additionally, if provided, statistical significance of individual studies was indicated by asterisks with * p ≤ 0.05, ** p ≤ 0.01 and *** p ≤ 0.001. Individual agreement between EP and CP as well as WEP and W’ was analysed by using participant data from the studies tables and – if only mean differences or correlations were reported – individual data were extracted from scatter plots. A corresponding documentation of these extractions is available as supplementary material (Appendix 1).

Meta-analyses were conducted for validating EP and WEP against CP and W’, respectively. Standardised mean difference (d_z_) was calculated as the ratio between mean difference and its corresponding standard deviation. Even though this approach is criticised for overestimating potential effects/differences in correlated samples, we intentionally used it in order to provide a conservative estimate on the potential to individually estimate CP and W’ by the 3-min all-out parameters (Lakens 2013). According to the literature, we provided small sample adjusted Hedge’s g_z_ and its corresponding variance for each study using CP and W’ (Lakens 2013; Borenstein 2023). A detailed description of all calculations is provided as Supplementary Material (Appendix 2).

### Synthesis methods

Studies were allocated to their synthesis group if the full text and the statistical analysis provided appropriate data corresponding to the aim of the study. If studies investigated both reliability and validity, they were allocated into both synthesis groups. If studies investigated both but only provided appropriate data for one synthesis group, they were only allocated to that respective group. The rationale for the exclusion was documented. If no data was reported for the required values, the column was filled with the next best value provided (e.g., the p-value). To ensure data comparability in the validity table, all r-values were converted into R²-values. In both tables, studies were sorted by PL with the highest one being on top of the list. Within each PL, studies were sorted alphabetically. Tabulations and illustrations on the data were performed in a customised spreadsheet (Microsoft EXCEL for Mac, Version 16.78.3, Microsoft, Redmond, WA, USA) that is available as supplementary material (Appendix 3).

Correlation and individual agreement between EP and CP as well as WEP and W’ were illustrated in a multi-panel Figure in which all studies are colour coded. If necessary, individual date were extracted from the studies plot and indicated in corresponding captions. Correlations to other physiological characteristics (e.g. V̇O_2max_ or individual time trial performance) were illustrated in Tables, but not included in syntheses. Since several studies used/compared different approaches to determine CP/W’ and/or EP/WEP, these were included as well. To avoid unwanted bias, results from a 90-s all-out test were not included in the synthesis (Barker et al. 2012), as this review explicitly targets the 3-min all-out test.

Meta-analysis was conducted by using a random effects model of pooled g_z_, since we regard every single study/effect as a random sample from the universe of exercise testing setting that vary in methodology. In order to compare this to fixed effect (singular) results, the corresponding pooled g_z_ was calculated as well (see Appendix 2 for details). Due to the small number of overall effects (k = 13 out of n = 8 studies), we refused to perform a sub-group analysis between methodological approaches that would have reduced statistical power substantially. Instead, all approaches were pooled as random-effects. In order to not only evaluate the precision of the effect, but also its dispersion, we calculated 95% prediction intervals (PIs) for random effects g_z_ (Borenstein 2023). Additionally, we calculated common language effect size (CLES) that indicates the likelihood that an individual finds that EP and WEP overestimates CP and W’, respectively.

### Reporting bias assessment

Risk of bias and publication bias were assessed by interpreting funnel plots illustrating the individual effects (g_z_) with respect to their corresponding standard error and symmetrical triangles to assist visual evaluation.

### Certainty assessment

Certainty was assessed in accordance to international guidelines of the GRADE domain for EP and WEP reliability and validity by evaluating risk of bias, inconsistencies, imprecision, indirectness and publication bias (Brignardello-Petersen and Guyatt 2025).

## Results

### Study selection

**Fig. 1:**
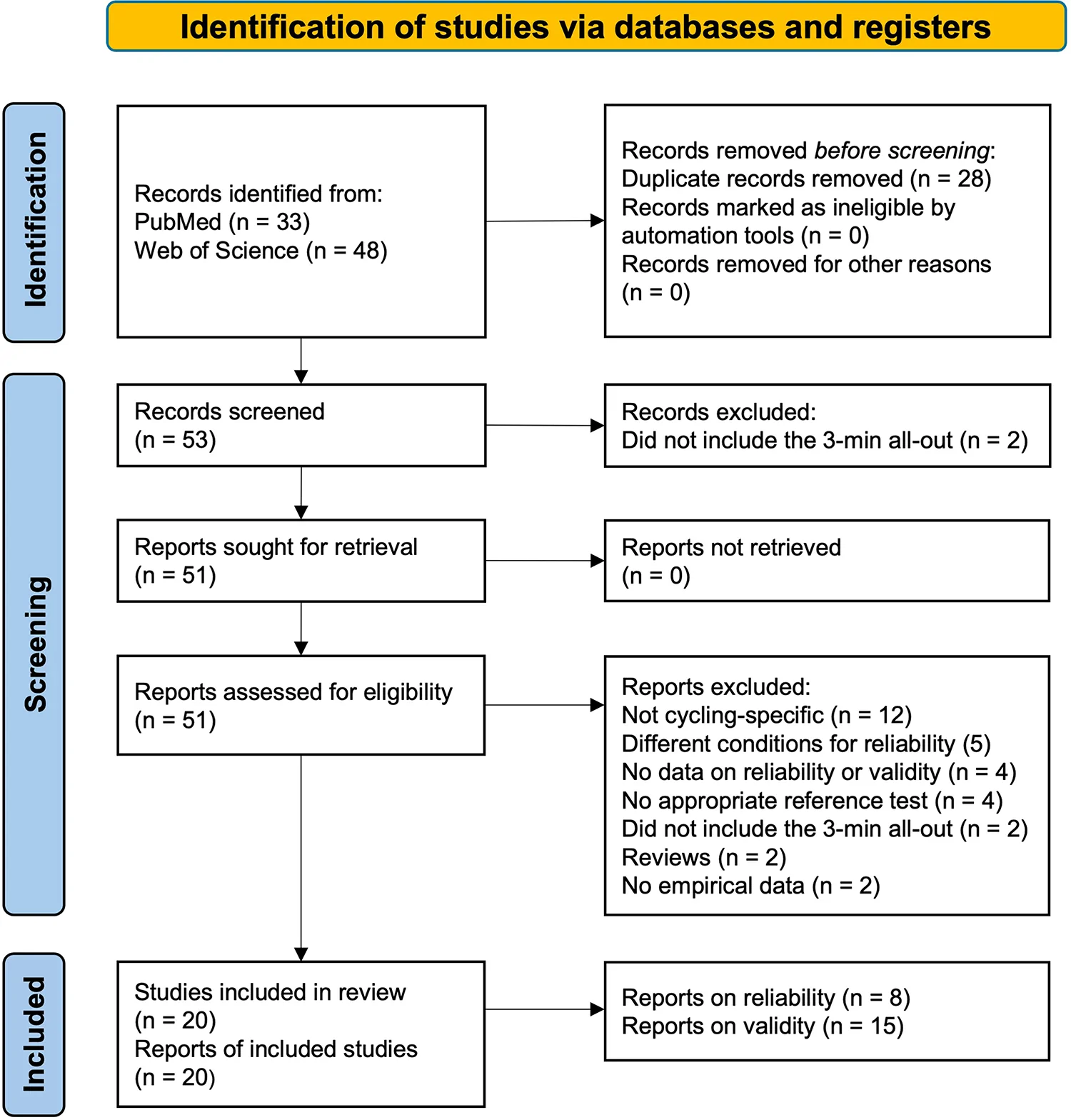
Flow diagram of study selection in accordance with PRISMA 2020 Statement

The PRISMA flow chart (Fig. 1) visualises the process of the literature search. Initial search yielded 81 records, with 33 identified using PubMed and 48 using Web of Science. After removing 28 duplicates, a total of 53 records remained for screening. Two records had to be removed due to clear irrelevance to the research topic. All remaining articles could be retrieved, resulting in 51 reports being assessed for eligibility. Twelve studies were excluded because of missing cycling specificity; containing six studies investigating in running (Gama et al. 2017, 2018; Kuo et al. 2017; Pettitt et al. 2012; Sousa et al. 2022; Tsai et al. 2025), two studies in arm cranking (Flueck et al. 2015; Flueck 2017), two studies in swimming (Tsai and Thomas 2017; Piatrikova et al. 2018), one study in rowing (Cheng et al. 2012) and one study in skiing (Fukuda et al. 2014). Secondly, five studies had to be excluded due to comparison of different conditions for reliability (Clark et al. 2013; Austad et al. 2013; Constantini et al. 2014; Dicks et al. 2016; Murgatroyd et al. 2014). Differences in test protocols and preconditions lead to a lack of standardisation and potentially impair results. Additionally, four studies had to be excluded due to missing data on reliability or validity regarding the 3-min all-out (Andersson et al. 2022; Mahoney et al. 2021; Olaya-Cuartero et al. 2023; Sreedhara et al. 2020). One of those studies provided respective data in an external file which was evaluated in another included study in more detail (Sreedhara et al. 2023). Four more articles were excluded because they did not use an appropriate reference test. Three of them validated the 3-min all-out with modified versions of the 3-min all-out itself (Kuo et al. 2021; Schulte et al. 2018; Tsai et al. 2024) and another study had little relation to endurance performance and was therefore considered inappropriate for validation (D’Hulst et al. 2024). Another two studies had to be excluded because they did not perform the 3-min as an all-out test (Hurst and Atkins 2006; Bhammar and Chien 2021). Furthermore, two investigations were excluded because they were (narrative) review articles (Pettitt et al. 2025; Chorley and Lamb 2020). Lastly, two more papers did not meet the inclusion criterion for collecting empirical data from the 3-min all-out test in cycling (Dekerle et al. 2008; Boillet et al. 2024).

Consequently, a total of *N* = 20 studies met all inclusion criteria and were included in this systematic review. Although Burnley et al. (2006) investigated both, reliability and validity, only the collected data on reliability could be used for the systematic evaluation in this review (Burnley et al. 2006). The data provided on validity was only concerning V̇O_2peak_ measurement instead of EP and WEP and therefore disregarded for the systematic approach. Similarly, some other studies provided several outcomes for the same quality criterion but not all of them were matching the study design and had to be partly excluded. This concerned a study that conducted two experiments with the second one being excluded for comparing data collected in a rested precondition with those collected in a pre-fatigued condition (Clark et al. 2018). 

### Study characteristics

The 20 studies included in this review varied in terms of sample characteristics, methodological approach and statistical evaluation. All studies were published between 2006 and 2025. Sample sizes ranged from 6 to 53 participants, covering all PLs (1–5). In only one study, more than 50% of the participants were female (Barker et al. 2012) suggesting to use the PL assessment for female participants (Decroix et al. 2016). Most studies conducted the 3-min all-out on a Lode Excalibur Sport (Burnley et al. 2006; Vanhatalo et al. 2007; Johnson et al. 2011; Clark et al. 2018; Goulding et al. 2021; Barker et al. 2012; Black et al. 2014; Wright et al. 2019; Zagatto et al. 2019; De Lucas et al. 2014) followed by CompuTrainer (Clark et al. 2016; Sreedhara et al. 2020; McClave et al. 2011), various types of smart trainer (Bartram et al. 2017; Maunder et al. 2022; Wilkins et al. 2025) or SRM ergometers (Dekerle et al. 2014; Karsten et al. 2013). They were using the linear mode (*n* = 13) either with a performance-based or a body mass based calculated resistance, the isokinetic mode (*n* = 4) or a freely geared mode if conducted on a smart trainer (*n* = 3). Few studies used ergometers in remote settings and some studies allowed participants to change gears during the test (Clark et al. 2016; Bartram et al. 2017; Maunder et al. 2022; Wilkins et al. 2025) (See Table 1).

**Table 1:** Results for risk of bias assessment

Study	(1)	(3)	(7)	(8)	(9)	(10)	(11)	(12)	(13)
category	B	V	V	R	V	B	V	B	B
Barker et al. (2012)	1	1	1	×	1	1	1	0	1
Bartram et al. (2017)	1	1	0	×	1	1	1	1	0
Black et al. (2014)	0	1	1	×	1	1	1	0	1
Burnley et al. (2006)	1	×	×	1	×	1	×	0	1
Clark et al. (2016)	1	1	0	×	1	1	1	0	1
Clark et al. (2018)	1	×	×	0	×	1	×	0	1
Clark et al. (2019)	0	1	0	×	1	1	1	0	1
De Lucas et al. (2014)	1	×	×	1	×	1	×	0	1
Dekerle et al. (2014)	0	1	0	×	1	1	1	0	1
Goulding et al. (2021)	0	1	0	×	1	1	1	1	1
Johnson et al. (2011)	1	×	×	0	×	1	×	0	1
Karsten et al. (2013)	1	1	0	1	1	1	1	0	1
Maunder et al. (2022)	1	1	1	1	1	1	1	0	0
McClave et al. (2011)	1	1	0	×	0	1	1	0	1
Sreedhara et al. (2023)	1	×	×	1	×	1	×	0	1
Vanhatalo et al. (2007)	1	1	0	×	1	1	1	0	1
Wilkins et al. (2025)	1	0	0	×	0	1	1	0	0
Wright et al. (2017)	0	1	0	1	1	1	1	0	1
Wright et al. (2019)	0	1	0	×	1	1	1	0	1
Zagatto et al. (2019)	1	0	0	×	1	1	1	0	1

(1) description of sample

(3) explanation of reference standard

(7) short time period between tests

(8) stability of variable being between repeated measures

(9) reference standard independent of index test

(10) test execution described in detail

(11) reference standard described in detail

(12) withdrawals explained

(13) statistical methods appropriate for purpose

1 = yes; 0 = no; **×** = not appropriate for study

*B = *both*, R = *reliability,* V = *validity

Fifteen studies dealt with validity and eight with reliability. Three studies included both criteria in their investigations (Maunder et al. 2022; Karsten et al. 2013; Wright et al. 2017). The reliability was assessed with test-retest designs completing at least two 3-min all-out tests (Table 2). Most studies compared both EP and WEP (Johnson et al. 2011; Maunder et al. 2022; Sreedhara et al. 2020; Clark et al. 2018; De Lucas et al. 2014; Wright et al. 2017) and only two studies solely provided values for EP (Burnley et al. 2006; Karsten et al. 2013). The validity of the test was primarily assessed against the gold standard of CP and Wʹ derived from multiple TTE trials, although V̇O_2_-values or time trial performance were used as well (Table 3). The three studies assessing both quality criterions without providing methodological limitations are presented in both tables. Separate risk auf bias ratings are available as Supplementary Material (Appendix 4) as well as additional information on study characteristics of studies assessing reliability and validity (Appendix 5 and 6, respectively).

**Table 2:** Methodological characteristics and outcomes for reliability studies

Study	*n *(f)	PL	Type of ergometer	Test mode	Test-retest duration	Outcome variable	Outcomes
Karsten et al. (2013)	13 (1)	4	SRM	iso kinetic	Within 21 days	EP	ICC = 0.97 (LoA: − 2 ± 37 W)
Burnley et al. (2006)	11 (2)	3	Lode Excalibur Sport	linear	Within 14 days	EP	ICC = 0.99*** (LoA: +1 ± 15 W)
Clark et al. (2018)	6 (0)	3	Lode Excalibur Sport	linear	Approximately 3 weeks	EP WEP	ICC = 0.99*** (LoA: − 3 ± 14 W) ICC = 0.99*** (LoA: − 0.4 ± 1.4 kJ)
De Lucas et al. (2014)	14 (0)	2	Lode Excalibur Sport	isokinetic	Within a period of 2–3 weeks	EP @ 60 rpm WEP @ 60 rpm EP @ 100 rpm WEP @ 100 rpm	ICC = 0.91, TE = 11.2 W ICC = 0.84, TE = 1.8 kJ ICC = 0.95, TE = 9.2 W ICC = 0.79, TE = 1.3 kJ
Johnson et al. (2011)	11 (5)	2	Lode Excalibur Sport	linear	n.r.	EP WEP	ICC = 0.89, TE = 21.4 W ICC = 0.76, TE = 27.5 W
Maunder et al. (2022)	53 (21)	2	Different smart trainer	freely geared	Each separated by 4–10 days	EP WEP	*r* = 0.97***, CV = 3.3% *r* = 0.90***, CV = 17.9%
Sreedhara et al. (2023)	7 (3)	2	CompuTrainer	linear	Within 2 weeks	EP WEP	ICC = 0.974, TE = 8 W ICC = 0.879, TE = 1.11 kJ
Wright et al. (2017)	12 (0)	2	Lode Excalibur Sport	linear AND isokinetic	n.r.	EP linear WEP linear EP isokinetic WEP isokinetic	ICC = 0.99***, CV = 1.2% ICC = 0.98***, CV = 5.4% ICC = 0.97***, CV = 1.9% ICC = 0.94***, CV = 8.4%

*CV* coeficcient of variability, *EP* endtest power, *f *females, *ICC* intra-class correlation coefficient, *LoA* 95% limits of agreement (± 1.96 SD), *n.r.* not reported, *PL* performance level, *rpm* revolutions per minute, *r* pearson’s correlation coefficient, *SRM* Schoberer Rad Messtechnik, *TE* typical error, *WEP* work above endtest power

* *p* ≤ 0.05; ** *p* ≤ 0.01; *** *p* ≤ 0.001

**Table 3:** Methodological characteristics and outcomes for validity studies

Study	*n *(f)	PL	Type of ergometer	Test mode	Criterion(s)	Outcome variable	Outcomes
Bartram et al. (2017)	6 (0)	5	Lemond Revolution Trainer	freely geared	CP and W´ of TTE tests of 1, 4, 6, 8 and 10 min	EP WEP	*P* < 0.001 *P* = 0.02
Karsten et al. (2013)	13 (1)	4	SRM	isokinetic	Calculated CP and W´ from TTE tests at 80, 100 and 105% MAP with 2 different equations	EP WEP EP WEP	Equation 1 LoA = 25 ± 48 W*** R² = 79,2%*** LoA = 3.27 ± 9.06 J R² = 18,5% Equation 2 LoA = 20 ± 41 W*** R² = 81%*** LoA = 1.43 ± 6.90 kJ R² = 23%
Black et al. (2014)	10 (n.r.)	3	Lode Excalibur Sport	linear	Time needed to complete a 16.1-km road TT	EP	R² = 69%***
Dekerle et al. (2014)	9 (0)	3	SRM	isokinetic	4–5 TTE tests between 3–15 min	EP 60 rpm WEP 60 rpm EP 100 rpm WEP 100 rpm	R² = 72.3%**; LoA = 14 ± 21 W R² = 50%* R² = 74%**; LoA = 15 ± 29 W R² = 2.6%
McClave et al. (2011)	21 (n.r.)	3	Compu-Trainer	linear	TTE at CP determined from 3MT	PP - EP	R² = 42,3%*
Vanhatalo et al. (2007)	10 (n.r.)	3	Lode Excalibur Sport	linear	Calculated CP and W´ from 5 TTE tests at 70 and 80%Δ, 100 and 105% V̇O_2peak_ and either 60%Δ OR 110% V̇O_2peak_	EP WEP	R² = 98,0% R² = 70,6%
Wright et al. (2019)		3	Lode Excalibur Sport	linear	Calculated CP and W´ from TTE tests at 80, 100 and 105% MAP	EP 3MT preferred WEP 3MT preferred EP 3MT −5 rpm WEP 3MT − 5 rpm EP 3MT + 5 rpm WEP 3MT + 5 rpm EP 3MT + 10 rpm WEP 3MT + 10 rpm	R² = 82,8%***; LoA = 30 ± 21 W*** R² = 46.2%*; LoA = −8.6 ± 10.1 kJ*** R² = 75.7%***; LoA = 36 ± 23 W*** R² = 25%; LoA = −7.7 ± 10.8 kJ* R² = 82.8%***; LoA = 23 ± 23 W*** R² = 22.1%; LoA = −9.4 ± 10.4 kJ** R² = 84,6%***; LoA = 11 ± 26 W R² = 17.6%; LoA = −8.9 ± 11.8 kJ*
Barker et al. (2012)	9 (6)	2	Lode Excalibur Sport	linear	Calculated CP and W´ from 3 TTE tests at individualized power outputs between 70–95% V̇O_2peak_	EP WEP	R² = 27% R² = 0.09%
Clark et al. (2016)	10 (3)	2	Compu- Trainer	linear	CP and W´ of TTE tests for 180/360/540 s	EP WEP	ICC = 0.972 ICC = 0.778
Clark et al. (2019)	14 (0)	2	Lode Excalibur Sport	linear	Calculated CP and W´ from TTE tests between 2–15 min of duration	EP WEP	R² = 83%*** R² = 27%
Goulding et al. (2021)	19 (0)	2	Lode Excalibur Sport	linear	CP and W` determined by 4 TTE bouts between 50% GET to V̇O_2max_ and 110% V̇O_2max_ (= duration between 2–15 min)	EP WEP	R² = 93%*** LoA = 6 W (−30, + 19 W) *P* = 0.034 LoA = 2.6 kJ (−5.8, + 11 kJ)
Maunder et al. (2022)	10 (2)	2	Wahoo Kickr (9) Taxc Neo 2 T (1)	freely geared	MMSS-V̇O_2_ MMSS-LA	EP EP	18% ±11*** 20%±12***
Wilkins et al. (2025)	18 (9)	2	Velotron Pro	freely geared	Prediction of %SmO2 by 3 exercise trials at 10% below EP, at EP and at 10% above EP	EP	R² = 77.4% LoA = −16.2 ± 18.6 W
Wright et al. (2017)	12 (0)	2	Lode Excalibur Sport	linear AND isokinetic	Calculated CP and W´from TTE bouts at 80, 100 and 105% MAP	EP linear WEP linear EP isokinetic WEP isokinetic	R² = 70.6%***; LoA = 30.22 ± 46.75 W** R² = 37.2%*; LoA = −9.27 ± 8.99 kJ*** R² = 67.2%**; LoA = 4.03 ± 29.68 W R² = 39.7%*; LoA = −7.12 ± 9.47 kJ***
Zagatto et al. (2019)	11 (0)	1	Lode Excalibur Sport	linear	MAOD AC[La⁻]+EPOCfast	AC AC	R² = 54.8%** LoA = −0.01 ± 1.02 L R² = 42.3%* LoA = −0.1 ± 1.23 L

*AC* anaerobic capacity, *CP* critical power, *EPOC* excess post-exercise oxygen consumption, *EP* endtest power, *f* females, *ICC* intra-class correlation coefficient, *La* lactate concentration, *LoA* limits of agreement, *MAOD* maximal accumulated oxygen deficit, *MAP* maximal aerobic power, *MMSS* maximal metabolic steady-state, *n.r.* not reported, *P*_max_ maximum power, *PL* performance level, *rpm* revolutions per minute, *R*² determination coefficient, *SRM* Schoberer Rad Messtechnik, *SmO*_2_ muscle (des-)oxygenation, *TT* time trial, *TTE* time to exhaustion, *V̇O*_2peak_ peak oxygen consumption, *W`* curvature constant, *WEP* work above endtest power

* *p* ≤ 0.05; ** *p* ≤ 0.01; *** *p* ≤ 0.001

### Risk of bias in studies

Across 148 items for risk of bias assessment rated independently, an instant agreement could be found in 99 cases (66.9%). For the remaining 49 assessments a consensus was found (Table 1). A detailed description of (dis-)agreement is provided as Supplementary Material (Appendix 4).

### Results of individual studies

#### Reliability studies

According to Koo and Li (2016) the ICC outcomes for EP and WEP were ‘good’ to ‘excellent’, ranging from 0.89 to 0.99 for EP and from 0.76 to 0.98 for WEP (Johnson et al. 2011; Sreedhara et al. 2023; De Lucas et al. 2014; Karsten et al. 2013; Wright et al. 2017). Studies that used r-values instead of ICCs reported strong correlations between 0.97 and 0.99 for EP and between 0.90 and 0.99 for WEP with all values being very highly significant (Burnley et al. 2006; Maunder et al. 2022; Clark et al. 2018). Two studies conducted four instead of two 3-min all-out tests (De Lucas et al. 2014; Wright et al. 2017). One study compared outcomes at 60 and 100 revolutions per minute (rpm) and provided ‘excellent’ ICCs of 0.91 and 0.95 for and ‘good’ ICCs of 0.84 and 0.79 for WEP, respectively (De Lucas et al. 2014). Another study compared the linear and the isokinetic test mode and showed ‘excellent’ values of 0.99 for EP and 0.98 for WEP at linear mode as well as 0.97 for EP and 0.94 for WEP using the isokinetic mode (Wright et al. 2017).

#### Validity studies

In 10 out of 15 studies, EP and WEP were validated against CP and Wʹ determined from multiple TTE bouts (Vanhatalo et al. 2007; Clark et al. 2016, 2019; Bartram et al. 2017; Goulding et al. 2021; Barker et al. 2012; Wright et al. 2017, 2019; Dekerle et al. 2014; Karsten et al. 2013). The R²-values for the validity within the ten studies ranged between 27% and 98% for EP and between 0.09% and 70.6% for WEP. The PL within these studies ranged from 2 to 5 and different test modes were chosen with linear mode used most (*n* = 6) followed by isokinetic mode (*n* = 2) and one study for freely geared as well as one study testing in both linear and isokinetic mode. Of the six studies applying a linear testing mode, two studies provided additional LoA ranging from 6 W (–30, + 19 W) (Goulding et al. 2021) to 36 ± 23 W (Wright et al. 2019) for EP and from 2.6 ± 8.4 kJ (Goulding et al. 2021) to −9.4 ± 10.4 kJ (Wright et al. 2017) for WEP. One of those studies investigated the impact of different cadences (preferred, −5 rpm, + 5 rpm and + 10 rpm) on the validity of the test (Wright et al. 2019). Outcome values for EP ranged from R² = 75.7% with LoAs of 36 ± 23 W for − 5 rpm to R² = 84.6% with LoAs of 11 ± 26 W for + 10 rpm. For WEP the outcome values ranged between R² = 22.1% with LoAs of −9.4 ± 10.4 kJ to R² = 46.2% with LoAs of −8.6 ± 10.1 kJ, respectively. Another linear test mode study provided ICCs of 0.972 for EP and 0.778 for WEP (Clark et al. 2016). The remaining three studies only reporting R²-values of EP and WEP showed values of 98% and 70.6% being the highest (Vanhatalo et al. 2007) and values of 27% and 0.09% being the lowest (Barker et al. 2012), respectively.

In the remaining 5 studies, EP was validated against 16.1-km time trial (TT) performance (Black et al. 2014), time to exhaustion at CP (McClave et al. 2011), maximal metabolic steady-state of lactate and V̇O_2_ (Maunder et al. 2022) and muscle oxygenation at, below and above EP (Wilkins et al. 2025).WEP was also validated against maximal accumulated oxygen deficit (Zagatto et al. 2019). The PL within these studies ranged from 1 to 3 and test modes using preferred (*n* = 3) or freely geared cadence (*n* = 3). Familiarisation was performed in 3 of these studies (Black et al. 2014; Maunder et al. 2022; Zagatto et al. 2019) (see Appendix 6). R² ranged between 18% and 77% for EP and 42–55% for whereas WEP. The highest correlations with EP were found to muscle oxygenation and TT performance (R² = 69%). WEP shared almost 60% with maximal accumulated oxygen deficit with mean deviation close to zero and LoAs of ±1.02 L.

### Results of syntheses

For the synthesis of the reliability outcomes, eight studies were included. All of them employed a test-retest design with the testing being completed within three weeks. The studies provided differences in participants regarding sample size (6–53 participants), age of participants (between 14.5 ± 0.3 and 40.5 ± 10.7 years), portion of female participants within sample (0–40%) and PL of participants (PL 2–4). Regarding type of ergometer and test mode, approximately 63% used the Lode Excalibur Sport in linear mode. The amount of laboratory visits ranged between three and eight. Within the visits, studies conducted between two and four 3-min all-out tests with six out of eight studies conducting an additional full familiarisation test. In 50% of the studies, V̇O_2_-values were measured as well. Considering warm-up and procedure thereafter, small differences in duration and intensity could be observed. Additionally, similar methods were used for statistical evaluation. Five studies provided ICCs for their results and three studies used r-values. Concerning the risk of bias assessment, in 35 out of 49 cases (71.4%), the items were assessed positively. All in all, the reliability studies provided sufficient comparability.

For the synthesis of the validity outcomes 15 studies were included. The studies varied in participants regarding sample size (6–50 participants), age (between 22 ± 2 and 42 ± 10), proportion of female participants within sample (0–67%) and PL (PL 1–5). Regarding type of ergometer and test mode the Lode Excalibur Sport as well as the linear mode were used for approximately half of the studies. Comparisons of the 3-min all-out with the gold standard of multiple TTE tests to determine CP and Wʹ was conducted by approximately half of the studies as well. The other studies used different criterions for validation including TT performance, steady states determined by physiological markers (V̇O_2_ and lactate) or percentage of muscle oxygen saturation (%SmO_2_). Considering warm-up and procedure thereafter, studies provided substantial differences in duration and intensity. Furthermore, 9 out of 15 studies conducted a full familiarisation test and 8 out of 15 studies measured additional V̇O_2_-values. For statistical evaluation, most studies reported R²-values with some providing additional LoAs. Three studies reported neither R²-values nor LoAs. Concerning the risk of bias assessment, in 65 out of 96 cases (67.7%), the items were assessed positively. Given the heterogeneity in methodological approaches, comparison of validity was challenging.

**Fig. 2:**
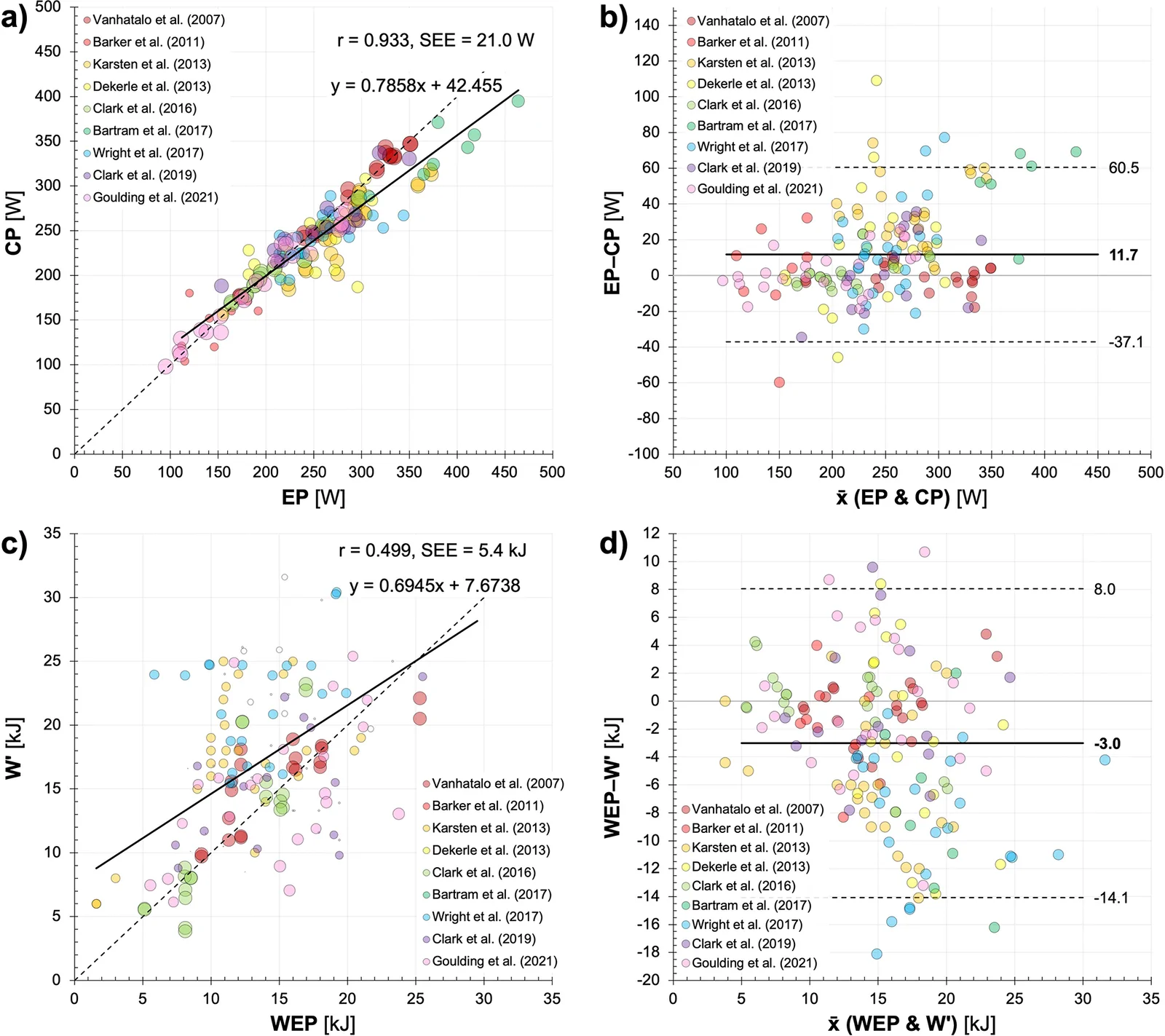
Pooled regression and Bland-Altman plots between CP and W’ and their surrogates **a** Scatterplot between EP and CP **b **Bland-Altman plot of the agreement between EP and CP **c **Scatterplot between WEP and W’ **d** Bland-Altman plot of the agreement between WEP and W’ *CP* critical power, *EP* endtest power, *r* pearson’s correlation coréfficient, *SEE* standard error of estimate, *WEP* work above endtest power, *W’* curvature constant

Pooling the studies’ individual results for EP and CP (including multiple approaches, N = 159), a high correlation (r = 0.993) with a standard error of estimate (SEE) of 21 W was observed (Fig. 2a). Mean difference was 11.7 W with LoA ranging from − 37.1 to + 60.5 W (Fig. 2b). Correlation between WEP and W’ was considerably lower (*r* = 0.499) with SEE = 5.4 kJ (Fig. 2c). Mean difference was − 3.0 kJ with high LoA (–14.1 to 8.0 kJ).

Pooled g_z_ of k = 15 effect sizes between EP and CP was found to be +0.408 (95%CI 0.196, 0.620) with PI ranging from 0.024 to 0.792. In comparison, fixed effect g_z_ was way closer to zero (0.041, 95%CI 0.003 to 0.079). However, neither CIs nor PI included zero indicating an overestimation of CP by using EP. Based on the studies average CLES, the likelihood for individual athletes to observe EP > CP is 62.1 ± 18.5%.

**Fig. 3:**
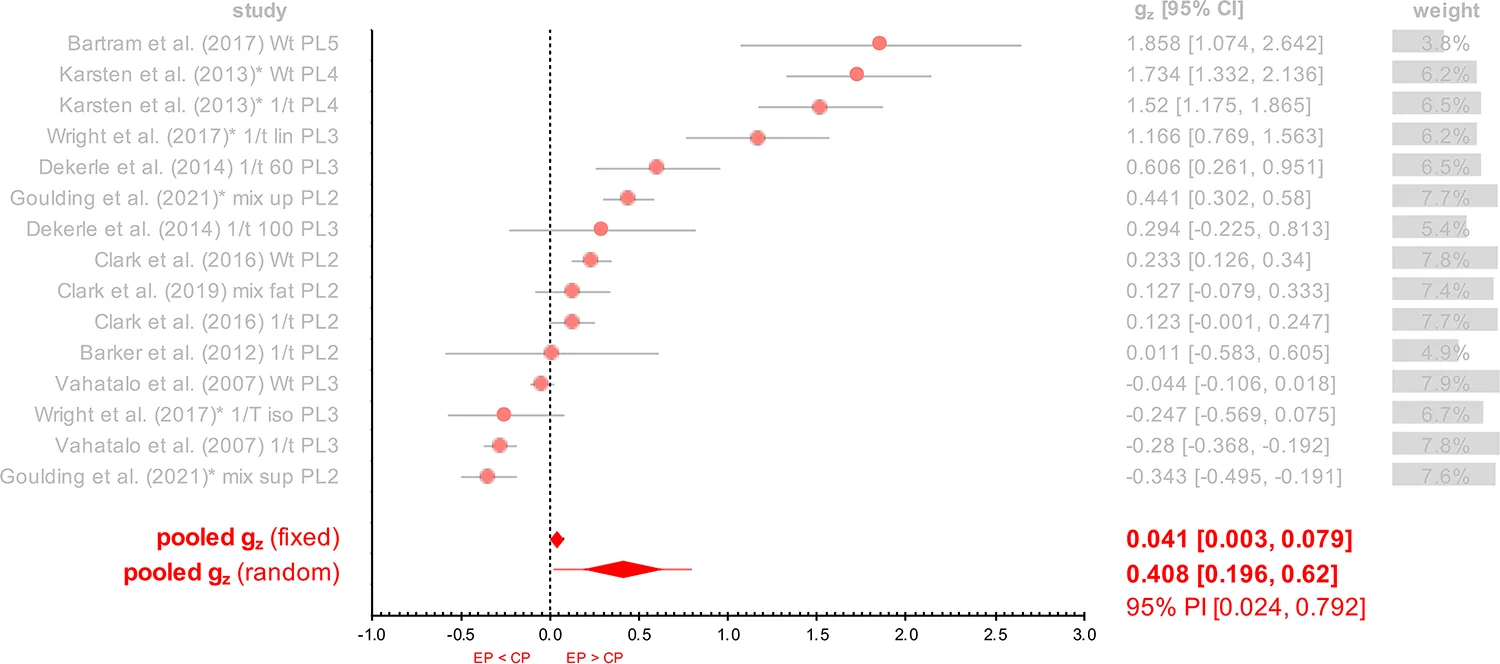
Forest plot on the agreement between EP and CP. 100 = 3-min all-out test performed at 100 rpm; 1/T = 1/T approach to determine CP and W’; 60 = 3-min all-out test performed at 60 rpm; *CI* confidence interval, *CP* critical power, *EP* endtest power, *fat* under fatigued conditions (2 h), *g*_z_ Heges g based on mean differences, *lin* linear mode during 3-min all-out test, *mix* best fit was used to determine CP and W’ (either WT or 1/T), *PI* prediction interval, *PL* performance level, *sup* supine position during trials, *up* upright position during trials, *WT* work-time approach to determine CP and W’ and *individual data extracted from Figures

**Fig. 4:**
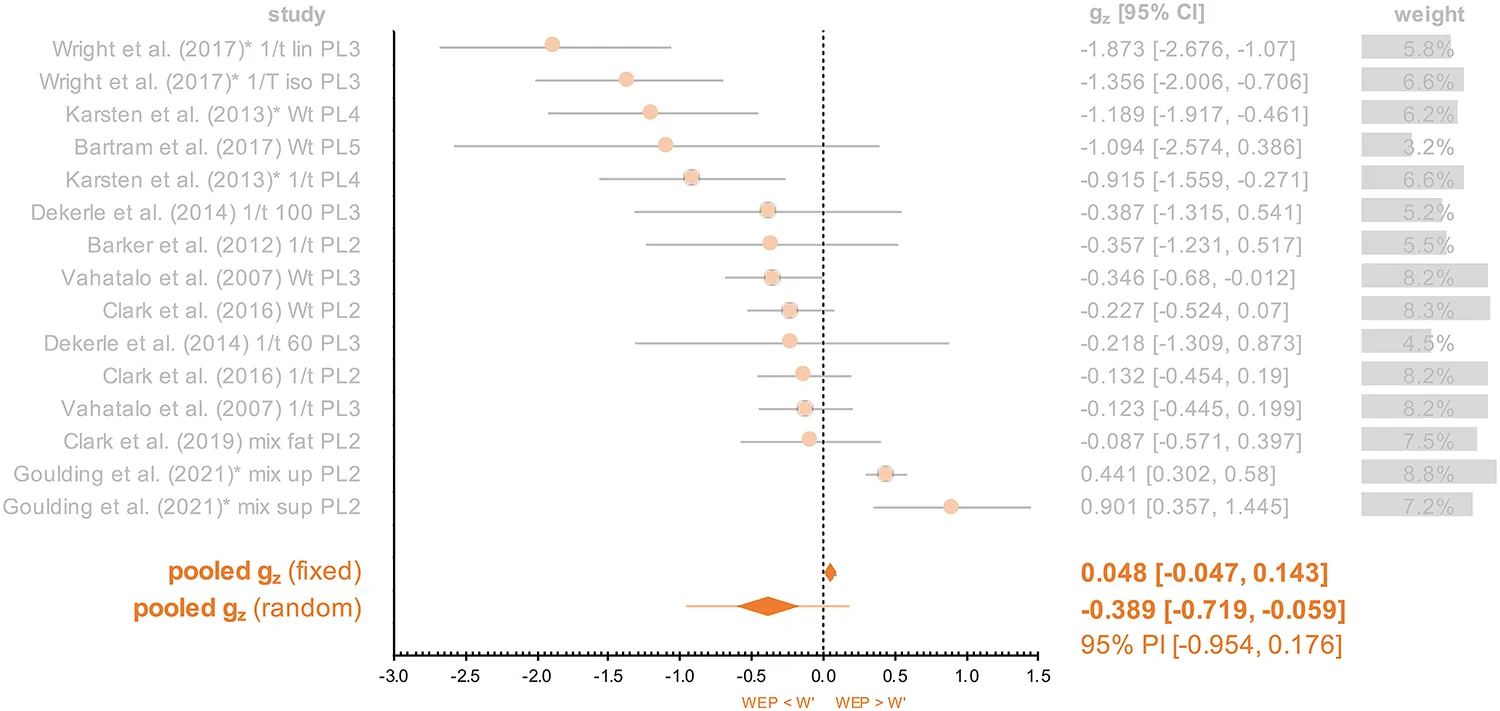
Forest plot on the agreement between WEP and W’. 100 = 3-min all-out test performed at 100 rpm; 1/T = 1/T approach to determine CP and W’; 60 = 3-min all-out test performed at 60 rpm; *CI* confidence interval, *fat* under fatigued conditions (2 h), *g*_z_ Hefges g based on mean differences, *lin* linear mode during 3-min all-out test, *mix* best fit was used to determine CP and W’ (either WT or 1/T), *PI* prediction interval, *PL* performance level, *sup* supine position during trials, *up* upright position during trials, *W’* curvature constant, *WEP* work above endtest power, *WT* work-time approach to determine CP and W’ and *individual data extracted from Figures

Pooled g_z_ of k = 15 effect sizes between WEP and W’ was found to be −0.389 (95%CI −0.719, −0.059) with PI ranging from −0.954 to 0.176. In comparison, fixed effect g_z_ was way closer to zero (0.048, 95%CI −0.047 to 0.143). Based on random effects CI, an underestimation of W’ by using WEP is likely, even though the true effect in the universe of exercise testing settings may include zero and the overestimation domain. Based on the studies average CLES, the likelihood for individual athletes to observe WEP > W’ is 35.6 ± 17.3% (Figs. 3 and 4).

**Fig. 5:**
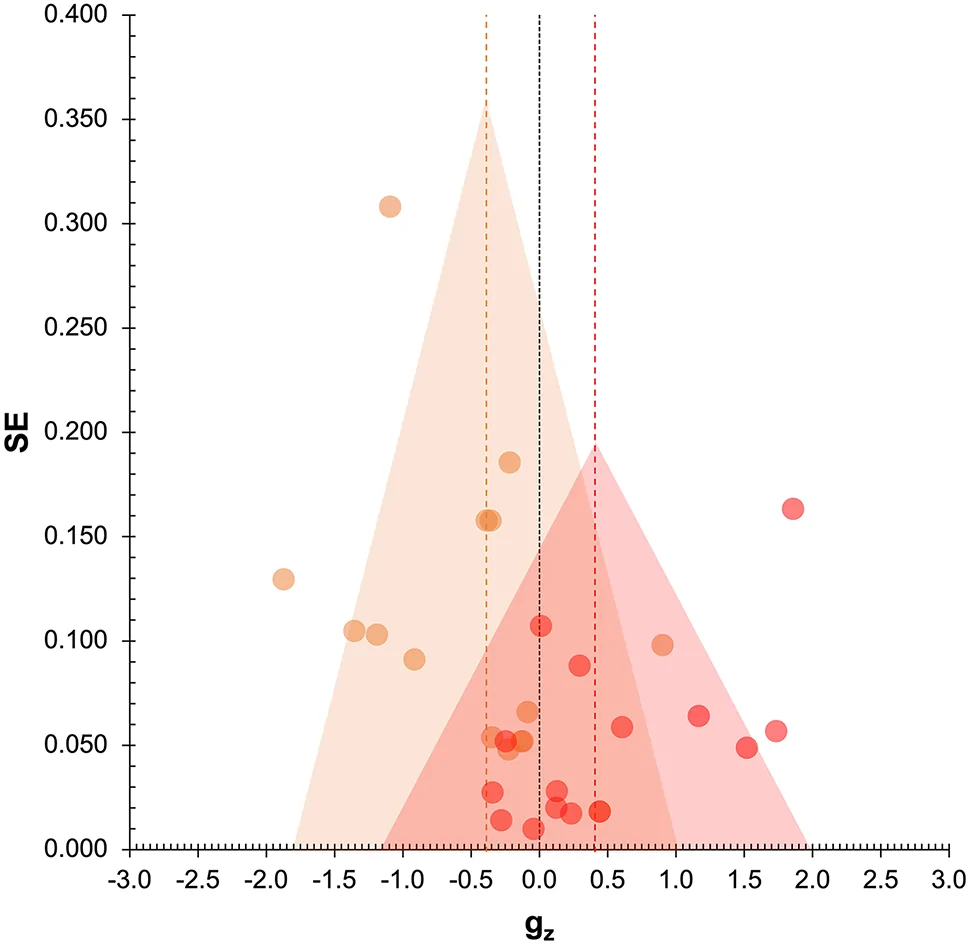
Funnel plot on publication bias for EP (red) and WEP (orange) agreement Note that shaded triangles are just illustrations to evaluate symmetry. *g*_z_ Heges g based on mean differences, *SE* standard error

Sensitivity analyses for publication bias of EP demonstrated a lack of symmetrical distribution for high g_z_ with small SE (Fig. 5). For WEP, funnel plot results were more heterogeneous but tended to be more symmetrical (Fig. 5). Certainty analysis indicated no concerns for EP reliability, moderate concerns for WEP reliability and high concerns regarding the validity of these measures (Table 4). Most concerning in the GRADE domain were inconsistency and imprecision, followed by risk of bias/publication bias, whereas indirectness did not seem to be an issue (except for WEP validity).

**Table 4.**
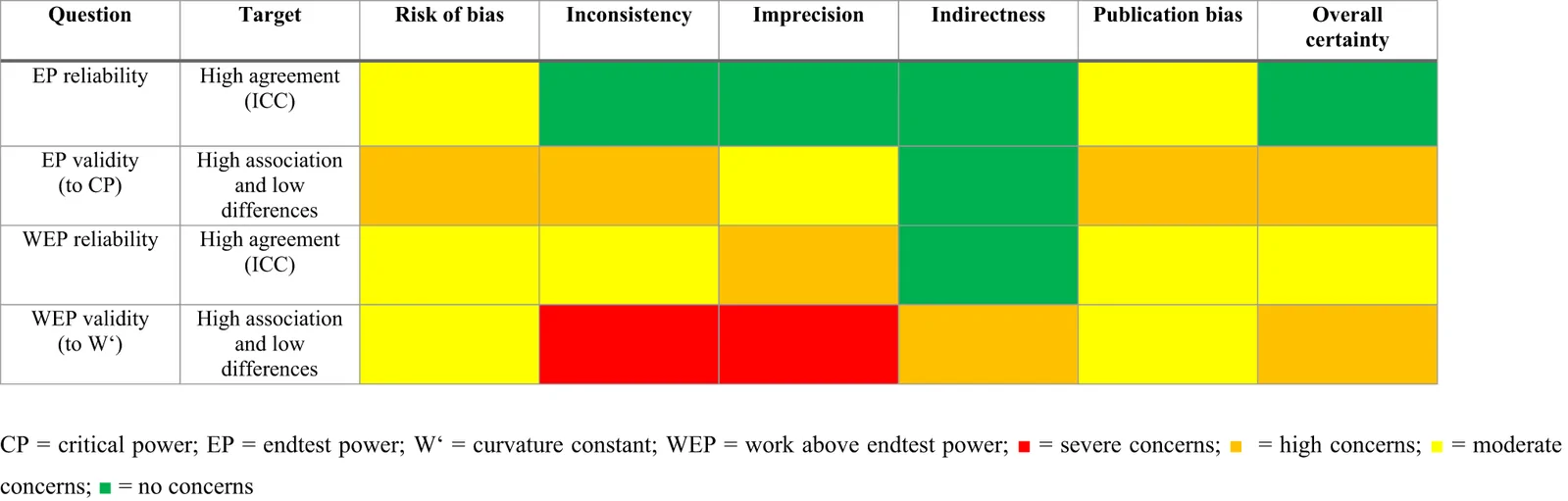
Assessing certainty about the reliability and validity of 3-min all-out test parameters

## Discussion

The aim of this systematic review and meta-analysis was to assess the reliability and validity of the 3-min all-out test in cycling and whether these are affected by methodological differences between studies. Our findings indicate that the reliability of EP and WEP is ‘good’ to ‘excellent’ irrespective of methodological characteristics if the tests are conducted within three weeks and familiarisation was provided. On the contrary, the validity of EP and WEP demonstrated heterogeneous outcomes across studies and is likely dependent on the test design of both 3-min all-out and CP-trials. Both parameters demonstrated high LoA to their counterparts (CP and W’, respectively) with considerable SEE, while EP and CP were highly correlated. Meta-analyses (random effects) found a systematic overestimation of CP (g_z_ = 0.408) and underestimation of W’ (–0.389) with rather huge dispersion of these effects (95%PI: 0.024 to 0.792 and −0.954 to 0.176, respectively). Especially for EP–CP, a possible publication bias favouring low deviations cannot be precluded. The following sections aim to differentiate the impact of changes in testing design for both reliability and validity and provide guidelines for appropriate testing setups.

### Endtest power (EP)

The study with the second worst outcomes (still ‘excellent’) only partially familiarised participants with the test (De Lucas et al. 2014). In accordance with earlier recommendations, it seems to be crucial for participants to be fully familiarised with the test protocol (Jones et al. 2010). Further, EP reliability were shown to be slightly better at higher cadences (100 rpm) compared to lower cadences (60 rpm) (De Lucas et al. 2014). Testing in isokinetic and linear test mode seemed to provide similar reliability outcomes (Wright et al. 2017). To investigate durability, a study examined the potential effects of prolonged endurance exercise on EP (Clark et al. 2018). Findings indicate that the 3-min all-out remains reliable after a standardised 2-h heavy-intensity exercise (Clark et al. 2018) which is likely transferable to other pre-fatigued condition as long as standardisation is provided.

Conclusions about the validity of 3-min all-out parameters are challenging as included studies demonstrated substantial, methodological differences with respect to CP-trials, calculation methods, 3-min all-out procedure, warm-up and participants. EP overestimated CP with highest effects observed in participants categorised as performance level 4 (well-trained) and 5 (professional) (De Pauw et al. 2013b). However, as nicely shown by Bartram et al. (2017), the validity of EP largely depends on the number and duration of CP-trials. Whereas CP derived from a “traditional” 5-effort approach (1, 4, 6, 8 and 10 min) resulted in substantially lower power compared to EP, the best agreement was found when using “3 carefully selected maximal efforts” of 1, 4 and 10 min (Bartram et al. 2017). This is in contrast to previous guidelines favouring efforts of 2–20 min (Vanhatalo et al. 2011; Jones et al. 2019b). Given that further studies in well-trained or elite cyclists concluded an overestimation of CP as well, the authors suggested an associated subject-group bias (Bartram et al. 2017). The present findings cannot support this suggestion. Firstly, the studies they refer to (McClave et al. 2011; Karsten et al. 2013; Bergstrom et al. 2013) involved participants classified between PL 2–4. Secondly, two PL 2 studies show an overestimation as well (Maunder et al. 2022; Wright et al. 2017). Both suggesting that the overestimation of CP determined from the 3-min all-out may stem from other factors than fitness level. More likely, the outcomes are affected by the choice of test mode or rather the choice of cadence.

Moreover, it has been argued that ergometer type (e.g. Lode vs. SRM) may be responsible for differences in EP-CP agreement (Karsten et al. 2013). In linear mode, participants work against a fixed resistance instead of a fixed cadence. Previous research showed that the 3-min all-out is sensitive to small changes in the preferred cadence (Wright et al. 2017; Vanhatalo et al. 2008b). The lack of information about the chosen cadence for the computation, mostly just reported as ‘preferred’, could have affected a variety of outcomes in studies employing linear mode. Another study on the sensitivity of the 3-min all-out concluded that a higher cadence leads to a lower EP and therefore a better agreement with CP (Wright et al. 2019). Moreover, since cadence is squared in the equation used for calculating the resistance (Burnley et al. 2006), it is sensitive to even small changes (Wright et al. 2019). In contrast to the original linear-factor equation (linear factor = power/cadence²), resistance can also be determined based on a fixed percentage of body weight (Bergstrom et al. 2012). Additionally, EP seems to be affected by the pedalling frequency with higher frequencies leading to lower EPs (Dekerle et al. 2014). Knowledge about the exact cadence used could have improved the interpretation. Furthermore, the isokinetic test mode was shown to provide better results compared to linear mode (Wright et al. 2017). Although similar intensities and consequently durations were used, trial-duration effects might impair results of calculated CP (Dotan 2022). Hence, the deviation between the outcomes may result from differences in CP determination by using the original protocol instead of the 3-min all-out test. In accordance with previous recommendations of four to five trials (Hill 1993), current evidence suggests that more TTE trials could lead to a higher agreement between EP and CP.

In all-out testing, performance is highly dependent on motivational aspects (Dotan 2022). Although all studies reported to encourage their participants during the 3-min all-out, intensity of encouraging may vary between studies. This could have an impact on the test outcome as most studies reported ‘strong verbal encouragement’ (Burnley et al. 2006; Vanhatalo et al. 2007; Sreedhara et al. 2023; Clark et al. 2018, 2019; Goulding et al. 2021; Barker et al. 2012; Wright et al. 2017, 2019; Zagatto et al. 2019; De Lucas et al. 2014; McClave et al. 2011; Wilkins et al. 2025; Dekerle et al. 2014; Karsten et al. 2013; Black et al. 2014), but others did not specify their intensity of encouragement (Johnson et al. 2011; Clark et al. 2016; Bartram et al. 2017) or did not report if encouragement was provided at all (Maunder et al. 2022). Although one of the studies that found an overestimation of CP used a method based on V̇O_2_-values and lactate instead of multiple TTE tests, it is also possible that missing encouragement resulted in using a pacing strategy to increase EP (Maunder et al. 2022). Even within the studies reporting ‘strong verbal encouragement’, the number of encouragers and/or their choice of words could impact motivation and therefore lead to different EPs. Despite the encouragement to an all-out strategy, it is suggested that pacing is still prevalent (Bartram et al. 2017). The underlying individual pacing choice may impact the test result (Johnson et al. 2011). Beyond normal motivational fluctuations, motivation could further be subsided if too many maximal effort visits are required (Johnson et al. 2011). This would lead to contradictory recommendations for the number of TTE trials. Subsequently, day-to-day differences could easily arise due to small changes in the aspects mentioned above.

In summary, performing the 3-min all-out test to derive EP should provide highly reliable outcomes provided that (a) familiarisation was performed, (b) strong verbal encouragement/music is provided throughout the test, (c) athletes are blinded for elapsed time and (d) isokinetic testing with cadences around 100 rpm is used. Performing the 3-min test to approximate an individual’s CP is not recommended, as the systematic overestimation largely depends on testing procedure and probably mediators, that have not yet been examined. Especially in PL4/5 athletes, EP seems to largely overestimate CP. Careful deciding for appropriate conditions with 3 maximal power efforts lasting 1–10 min may improve EP-CP agreement.

### Work above endtest power (WEP)

Similar to EP, it is unlikely that WEP reliability is impaired by test mode, type of ergometer or PL. Given that studies reported a test-retest duration of three weeks at the highest, it is possible that testing intervals that go beyond that might be inappropriate and impair testing results. Notably, the study that investigated the oldest participants (42 ± 10 years) resulted in low ICCs for WEP. Further, the same study conducted four 3-min all-out tests in total and showed that more trials lead to better ICCs for WEP at each trial (Sreedhara et al. 2023). These present findings in cycling provide slightly better results compared to a 3-min all-out conducted in other sports like running (Sousa et al. 2022) or rowing (Cheng et al. 2012). Further, prior sprint exercise reduces WEP in contrast to EP (Vanhatalo and Jones 2009).

Compared to EP, WEP disagreements to its counterpart (W’) yielded substantially higher dispersion with lower overall correlation. Regarding the methodological characteristics, cadence seems to have the greatest impact on WEP outcome. The findings indicate that WEP underestimates Wʹ at cadences that are higher than preferred (Francis et al. 2010). Further, lower cadences lead to better estimates or even overestimations of Wʹ (Goulding et al. 2021; Wright et al. 2019; Clark et al. 2019; Dekerle et al. 2014; Karsten et al. 2013). In particular, one study demonstrated vast differences in outcomes for isokinetic testing at 100 rpm compared to 60 rpm (Francis et al. 2010). Additionally, another study indicates that even small changes of cadence in linear mode from −5 to +10 rpm could lead to different outcomes (Wright et al. 2019), which is supported by one of the original studies (Vanhatalo et al. 2008b). The same study concluded that cadences near to the participants’ preferred one provide better values for WEP than those further away (Wright et al. 2019). Assuming a preferred cadence of 80–90 rpm for most participants (Burnley et al. 2006), this finding would be contradictory with the isokinetic studies showing better results for WEP if cadence was substantially lower (60 rpm) than preferred (Dekerle et al. 2014). These findings underline the importance of reporting the participants’ preferred cadence in more detail.

Only one study focused on the determination of anaerobic capacity by the 3-min all-out and compared two methods (Zagatto et al. 2019). Their comparison of determined anaerobic capacity using a different method with two well established methods, revealed valid results. Thus, summing the fast component of excess post-exercise oxygen consumption and the oxygen equivalent for energy provided by blood lactate accumulation (Bertuzzi et al. 2010) could be a valid method for estimating anaerobic capacity during a 3-min all-out. However, another study concludes that neither maximal accumulated oxygen deficit nor Wʹ can be fully explained by the anaerobic energy production, suggesting further factors to contribute (Muniz-Pumares et al. 2017). To answer the question if anaerobic capacity can be measured indirectly, future studies are warranted.

Given the mathematical connection of CP and W’ (Burnley et al. 2006), it is not surprising, that EP and WEP tended towards over- and underestimation, respectively. In practice, this observation is confirmed as endurance training increases CP but tends to decrease Wʹ (Jones et al. 2010). The authors suggested a subject-group bias based on training status (Bartram et al. 2017), which cannot be confirmed by the present review, because Wʹ seems to be underestimated at any PL (Wright et al. 2017, 2019; Clark et al. 2019; Dekerle et al. 2014). In contrast to PL, age could have a greater impact, as adolescents provide the lowest outcomes for Wʹ (Barker et al. 2012), likely due to a reduced anaerobic fitness (Muniz-Pumares et al. 2019).

In summary, WEP can be determined reliably – even after performing extensive exercise in terms of durability testing. As with EP, prior familiarisation, strong verbal encouragement and high isokinetic cadences (around 100 rpm) are recommended to improve accuracy. We strongly advise against estimating W’ by means of WEP since the large effect size, high dispersion and low correlation hinders this. Instead, WEP can be seen as a distinct proxy of ‘anaerobic capacity’ (Heck et al. 2003). However, compared to EP, the sensitivity of WEP and potential for estimation errors is higher.

### Additional parameters (P_max_ and V̇O_2max_)

Given that the 3-min all-out should be identical to a sprint test, it seems feasible to easily extract P_max_ as well. However, the 3-min all-out was designed to reach its nadir of pedalling frequency at the end of the test near the participants’ preferred cadence (Clark et al. 2013). To ensure that preferred cadence is still achieved, the fixed resistance needs to be low enough. For the determination of P_max_, high resistances of 7.5% of body mass or more are required (Dekerle et al. 2008). Due to the power-velocity relationship, high resistances would be suboptimal for the determination of other parameters (Dekerle et al. 2008) and would likely cause premature fatigue (Dicks et al. 2016). Therefore, it is not possible to reach real P_max_ within a 3-min all-out test conducted with a fixed resistance (linear mode). Moreover, peak power is not only dependent on resistance but also pedalling frequency (Sargeant et al. 1981). P_max_ is attained at around 110 rpm (Sargeant et al. 1981), which likewise requires isokinetic testing (De Lucas et al. 2014; Haase et al. 2024). So far, no study conducted the 3-min all-out using pedalling frequencies above 100 rpm, making it unlikely for P_max_ to be attained during the test. On the other hand, if cadence is further increased, it may be suboptimal for other variables like EP (De Lucas et al. 2014).

Originally, the 3-min all-out was developed to not only determine the maximal metabolic steady-state but to measure V̇O_2peak_ as well (Dekerle et al. 2008; Burnley et al. 2006). In isokinetic mode, the measurement of V̇O_2peak_ is unaffected by a change in cadence (60 and 100 rpm) and reported as highly reliable (De Lucas et al. 2014). This finding is in accordance with studies conducted in running (Sousa et al. 2022). Furthermore, it is reported to be sensitive to changes in maximal markers of aerobic function (Vanhatalo et al. 2008a). However, other studies have shown that V̇O_2max_ is not fully attained during a 3-min all-out (Vanhatalo et al. 2007; McClave et al. 2011; Hanson et al. 2022; Sperlich et al. 2014). Thus, to attain true V̇O_2max_, it is strongly recommended to conduct an additional square wave bout at 10% above determined CP shortly, subsequent to the 3-min all-out (Clark et al. 2013; Johnson et al. 2011). The further rise in V̇O_2_ would additionally confirm that EP is in close proximity to CP (Clark et al. 2013).

### Limitations

As pointed out earlier, comparability of the included studies was challenging due to multiple aspects, such as variety in the methodological approach and statistical evaluation. Furthermore, disparities in cadence/resistance, encouragement of participants and missing familiarisation may lead to varying results. As objectivity is a cornerstone of scientific research, it must be critically appraised as well. Strong verbal encouragement, as provided in most of these studies, may lack objectivity and/or standardisation and could therefore affect the participants’ motivational state. Moreover, studies used different durations and intensities for warm-ups and for the procedures thereafter. Improved reporting and standardisation of these procedures may improve the comparability between studies. Furthermore, the included studies differed vastly in sample size with some studies investigating only a few participants questioning the credibility of their results (Bartram et al. 2017; Sreedhara et al. 2023; Clark et al. 2018). Also, several studies did not report the sex of their participants leading to impaired interpretability (Vanhatalo et al. 2007; Black et al. 2014; McClave et al. 2011). Given that most values for V̇O_2max_ are not reported separately for male and female participants, the allocation to their specific PLs was challenging. This could dampen the potential subject-group bias suggested by one study (Bartram et al. 2017). In general, studies should report the PL of their participants separately according to guidelines for females and males (Decroix et al. 2016; De Pauw et al. 2013a). Otherwise, fitness of participants could be over- or underestimated, like in one study that described their participants as ‘elite cyclists’ but were allocated to PL 3 in this review (McClave et al. 2011). Additionally, it should be pointed out that studies clearly distinguish between an ‘all-out’ effort and a TT, as the latter involves a pacing strategy for attaining the highest average power/speed. Mixing both terms leads confusion and hinders appropriate test instructions (Andersson et al. 2022).

Meta-analyses provided in this article based on random effects model that takes individual study differences into consideration (Borenstein 2023). Effect size estimates used here are fairly conservative and tend to inflate with higher mean differences and strong correlations (Lakens 2013). This implies that effect sizes highlighted in Figs. 3 and 4 might be higher when compared to repeated measures determination, but the choice was intensional to provide most realistic estimates for individual athletes (Lakens 2013). It has to be noted that meta-analyses included all available effects with most studies reporting more than one approach. These additional approaches were weighted as individual datasets which has been discussed to skew effects in a previous critique on meta-analyses in strength and conditioning research (Kadlec et al. 2022). While we are fully aware about this issue in examining the effect of training interventions, we believe that using half samples for weighted effects is worse for the overall interpretation than doubling them as individual effects. In that respect, they represent different approaches that are available when performing these exercise tests. However, hierarchical weighting considering the origin of additional approaches within studies may be performed to ensure that our findings are not biased. Publication bias cannot be precluded for the results of individual studies – especially with respect to EP. Consequently, pre-registered protocols and trials may increase the probability of studies to be accepted when significant differences to gold-standard methods is observed. Lastly, validating EP and WEP by means of CP and W’ may be too simplistic, as the critical power model has been criticised in the literature (Dotan 2022; Gorostiaga et al. 2022). Other parameters (e. g. maximal lactate steady-state, MLSS) may provide a more accurate validation for EP (Billat et al. 2003; Denadai et al. 2005; Niemeyer et al. 2022). However, it is unlikely that EP and MLSS demonstrate a better agreement since CP already tends to overestimate MLSS (Caen et al. 2024; Jones et al. 2019b). In that respect, alternative calculation models like the 3-parameter model to determine CP should be examined in future studies (Morton 1996).

### Future directions

This current review aimed to evaluate the reliability and validity of the 3-min all-out in cycling, as well as to compare different methodological approaches to conduct this test. As the 3-min all-out is a test with great potential, it is conducted in other sports as well. Future studies should aim to optimise the methodological approach beyond cycling. Within the cycling studies, the present review demonstrated an impact of chosen cadence on test results. Especially for studies using the linear-factor equation, future research should aim to reduce the impact of a non-standardised parameter as preferred cadence. This could be done by transforming the current equation or ideally excluding preferred cadence entirely. Since it was shown that higher cadences lead to better results for EP, future research should investigate to what extent increasing cadence could improve outcomes. Moreover, the effect of different warm-up protocols needs to be examined systematically. Additionally, we found that female participants are underrepresented in the included studies that demonstrates the need to focus on this population. The variability of EP, WEP and P_max_ across the menstrual cycle is another phenomenon worth examining.

### Practical application

In general, it is recommended to prioritise one target parameter as it is currently not possible to yield valid results for all derived parameters at once. Furthermore, the methodological approach to determine that parameter could be specified depending on additional desired parameters without impairing the validity of the prioritised one. As for now, we recommend conducting a 3-min all-out test in cycling as follows: isokinetic mode, at high cadence (≥ 100 rpm) and after performing a full familiarisation test. In accordance with earlier testing criteria (Jones et al. 2010), additional aspects, such as high motivational state, appropriate cadence throughout the test, and the absence of any time-based feedback should be considered.

If V̇O_2peak_ is targeted, an additional square wave bout 10% above EP should be conducted. If only linear mode testing is feasible, the effect of preferred cadence should be reduced. Thus, determination of resistance through percentage of body mass is preferred and enables a real single-visit protocol. For most participants 3–4.5% body mass will provide acceptable results. Since these given workloads are fairly low concerning a sprint test, this protocol may result in an underestimation of P_max_. Further, it is possible to conduct the 3-min all-out test in remote settings, but results could be impaired if no encouragement is provided.

## Conclusion

The 3-min all-out test in cycling provides a useful and reliable tool for determining performance indices of power and work in a short amount of time. However, EP and WEP tend to over- and underestimate their corresponding counterparts (CP and W’) with large dispersion that depends on the methodological approaches. Even though higher cadences during the 3-min all-out seem to improve the agreement between EP and CP, we advise against calculating CP and W’ based on linear regression. Instead, EP and WEP can be used as separate parameters of metabolic/physiological profiling. Testing results are impaired if no full familiarisation test and/or strong verbal encouragement is performed. 

## Supplementary Information

Below is the link to the electronic supplementary material.

Supplementary Material 1

Supplementary Material 2

Supplementary Material 3

Supplementary Material 4

Supplementary Material 5

Supplementary Material 6

## Abbreviations

CP: Critical power [W]
EP: Endtest power [W]
g_z_: Hedge’s g based on mean difference and the corresponsing standard deviation
ICC: Intraclass correlation coefficient
LoA: Limit of agreement (±1.96 standard deviation)
PI: Prediction interval
PL: Performance level
P_max_: Maximum power [W]
r: Pearson’s correlation coefficient
rpm: Revolutions per minute
R²: Determination coefficient (%)
SEE: Standard error of estimate
SRM: Schoberer Rad Messtechnik
TTE: Time to exhaustion [min]
V̇O_2max_: Maximal oxygen uptake [ml/min/kg]
V̇O_2peak_: Peak oxygen uptake [ml/min/kg]
WEP: Work above endtest power [J]
Wʹ: Curvature constant [J]
