# Supplementary material for: The 3-min all-out test in cycling: a systematic review and meta-analysis on its reliability and validity
Source: Eur J Appl Physiol. 2026 Jul 21;126(9):4673–91. doi: 10.1007/s00421-026-06356-w (PMC13541879; doi:10.1007/s00421-026-06356-w)
Supplement: Supplementary file 1 [file 421_2026_6356_MOESM2_ESM.docx]

**Appendix 2 – Supplementary methods on effect size and synthesis calculations**

All calculations were done in a customised spreadsheet (Microsoft EXCEL, Version 16.78.3 for Mac, Microsoft, Redmond, WA, USA). That is also uploaded as supplementary material. Effect-sizes were calculated as Hedge’s g (g_z_) by utilising the ratio of mean difference (M_diff_), its corresponding standard deviation (SD_diff_) – also known as Cohen’s d_z_ – and a correction factor for small (n<20) samples (J) in accordance to Equation (1):

g_z_ = $\text{d}\text{z}\text{ ∙ J = }\frac{\text{M}\text{diff}}{\text{SD}\text{diff}}\text{ ∙ (1–}\frac{\text{3}}{\text{4n–9}}\text{)}$	(1)

where d_z_ = standardised mean difference; g_z_ = Henge’s g based on standardised differences; J = correction factor for small (n <20 ) samples; M_diff_ = mean difference between approaches (EP–CP and WEP–W’); n = sample size and SD_diff_ = corresponding standard deviation to differences between approaches.

The corresponding variance (V_z_) was calculated by utilising the variance of standardised mean difference (d_z_) (V_d_) and a correction factor for repeated measures and includes their correlation:

V_z_ = V_d_ $\text{∙}$ J^2^ = ($\frac{\text{1}}{\text{n}}\text{∙}\frac{\text{d}\text{z}\text{2}}{\text{2n}}\text{) ∙ 2(1–r) ∙ J}\text{2}$	(2)

where d_z_ = standardised mean difference; g_z_ = Henge’s g based on standardised differences; J = correction factor for small (n <20 ) samples; M_diff_ = mean difference between approaches (EP–CP and WEP–W’); n = sample size; r = actual correlation between EP and CP or WEP and W’, respectively; SD_diff_ = corresponding standard deviation to differences between approaches; V_d_ = variance corresponding to the effect indicated by d_z_ and V_z_ = variance corresponding to the effect of g_z_.

To indicate the probability of overestimating CP or W’ when performing the 3-min all-out, common language effect size (CLES) was calculated in accordance to Equation (3):

CLES = $\text{Φ}\text{(}\frac{\text{M}\text{diff}}{\text{ SD}\text{diff}\text{ ∙ }\sqrt{\text{2}}}\text{)}$	(3)

where Φ = percentile for normal distribution based on a standardises z-score; CLES = common language effect size; M_diff_ = mean difference between approaches (EP–CP and WEP–W’); SD_diff_ = corresponding standard deviation to differences between approaches.

# Pooled g_z_ for fixed effect was calculated as the ratio between the sum of individual studies weighted effects and the sum of all weights in accordance to Equation (4):

pooled g_z_ (fixed effect) = $\frac{\sum_{\text{i}}^{\text{k}} \text{(g}\text{z}\text{/V}\text{z}\text{)}}{\text{ }\sum_{\text{i}}^{\text{k}} \text{(1/V}\text{z}\text{)}}$	(4)

where g_z_ = Henge’s g based on standardised differences; k = number of effects involved (k = 13); i = individual study and V_z_ = variance corresponding to the effect of g_z_.

# The corresponding variance (V_g_) of pooled fixed effect g_z_ was calculated in accordance to Equation (5):

pooled V_g_ (fixed effect) = $\frac{\text{1}}{\sum_{\text{i}}^{\text{k}} \text{(1/V}\text{z}\text{)}}$	(5)

where k = number of effects involved (k = 13); i = individual study and V_z_ = variance corresponding to the effect of g_z_.

Homogeneity (Q) was calculated by also using the sum of squared g_z_ divided by V_z_ as indicated in Equation (6):

Q = $\sum_{\text{i}}^{\text{k}} \text{(g}\text{z}\text{2}\text{/V}\text{z}\text{)}\text{–}\frac{\sum_{\text{i}}^{\text{k}} \text{(g}\text{z}\text{/V}\text{z}\text{)}\text{2}}{\sum_{\text{i}}^{\text{k}} \text{(1/V}\text{z}\text{)}}$	(6)

where g_z_ = Henge’s g based on standardised differences of individual studies; i = individual study; k = number of effects involved (k = 13); Q = homogeneity indicator and V_z_ = variance corresponding to the effect of g_z_.

Between study variance (τ^2^) was calculated by utilising Q in accordance to Equation (7):

τ^2^ = $\frac{\text{Q}\text{–}\text{(k}\text{–}\text{1)}}{\sum_{\text{i}}^{\text{k}} \text{(1/V}\text{z}\text{)}- \frac{\sum_{\text{i}}^{\text{k}} \text{(1/V}\text{z}\text{)}\text{2}}{\sum_{\text{i}}^{\text{k}} \text{(1/V}\text{z}\text{)}}}$	(7)

where g_z_ = Henge’s g based on standardised differences of individual studies; i = individual study; k = number of effects involved (k = 13); Q = homogeneity indicator and V_z_ = variance corresponding to the effect of g_z_.

In order to perform a random effects model meta-analysis and calculate the pooled effect and variance for g_z_, Equation (4) and (5) were adapted by adding τ^2^ to the individual study variance (V_z_+τ^2^) as highlighted in Equations (8) and (9):

pooled g_z_ (random effects) = $\frac{\sum_{\text{i}}^{\text{k}} \text{(g}\text{z}\text{/V}\text{z+}\text{τ}\text{2}\text{)}}{\text{ }\sum_{\text{i}}^{\text{k}} \text{(1/V}\text{z+}\text{τ}\text{2}\text{)}}$	(8)

where g_z_ = Henge’s g based on standardised differences; k = number of effects involved (k = 13); i = individual study; V_z_ = variance corresponding to the effect of g_z_ and τ^2^ = between study variance.

# The corresponding variance (V_g_) of pooled random effects g_z_ was calculated as Eq. (9):

pooled V_g_ (random effects) = $\frac{\text{1}}{\sum_{\text{i}}^{\text{k}} \text{(1/V}\text{z}\text{+}\text{τ}\text{2}\text{)}}$	(9)

where k = number of effects involved (k = 13); i = individual study; V_z_ = variance corresponding to the effect of g_z_ and τ^2^ = between study variance.

Finally, 95% prediction interval (PI) for pooled random effects g_z_ was calculated by using critical value from t-distribution (t) and τ^2^ in accordance to Equation (10):

PI = ± t(k–1) $\text{∙ }\sqrt{\sum_{\text{i}}^{\text{k}} \text{(1/V}\text{z+}\text{τ}\text{2}\text{)}\text{+}\text{τ}\text{2}}$	(10)

where k = number of effects involved (k = 13); i = individual study; PI = prediction interval (95%); V_z_ = variance corresponding to the effect of g_z_ and τ^2^ = between study variance.
