# Supplementary material for: The 3-min all-out test in cycling: a systematic review and meta-analysis on its reliability and validity
Source: Eur J Appl Physiol. 2026 Jul 21;126(9):4673–91. doi: 10.1007/s00421-026-06356-w (PMC13541879; doi:10.1007/s00421-026-06356-w)
Supplement: Supplementary file 2 [file 421_2026_6356_MOESM4_ESM.docx]

**Appendix 4 – Risk of bias assessment of both raters before consensus**

| Study | (1) | (3) | (7) | (8) | (9) | (10) | (11) | (12) | (13) |
| --- | --- | --- | --- | --- | --- | --- | --- | --- | --- |
| category | **B** | **V** | **V** | **R** | **V** | **B** | **V** | **B** | **B** |
| Barker et al. (2011) | 1 | 1 | 1 | × | 1 | 1 | 1 | 2 | 2 |
| Bartram et al. (2017) | 1 | 1 | 0 | × | 1 | 2 | 2 | 1 | 0 |
| Black et al. (2014) | 2 | 1 | 1 | × | 2 | 1 | 2 | 0 | 1 |
| Burnley et al. (2006) | 1 | × | × | 1 | × | 1 | × | 2 | 1 |
| Clark et al. (2016) | 1 | 2 | 0 | × | 1 | 2 | 2 | 0 | 2 |
| Clark et al. (2018) | 1 | × | × | 2 | × | 1 | × | 0 | 2 |
| Clark et al. (2019) | 2 | 1 | 0 | × | 1 | 1 | 1 | 0 | 1 |
| De Lucas et al. (2013) | 2 | × | × | 1 | × | 1 | × | 0 | 1 |
| Dekerle et al. (2013) | 2 | 2 | 0 | × | 2 | 1 | 2 | 0 | 1 |
| Goulding et al. (2021) | 2 | 1 | 0 | × | 1 | 1 | 1 | 1 | 1 |
| Johnson et al. (2011) | 1 | × | × | 2 | × | 1 | × | 0 | 1 |
| Karsten et al. (2013) | 1 | 1 | 2 | 1 | 1 | 1 | 2 | 0 | 1 |
| Maunder et al. (2022) | 1 | 2 | 1 | 1 | 1 | 1 | 1 | 0 | 2 |
| McClave et al. (2014) | 1 | 1 | 2 | × | 0 | 1 | 2 | 0 | 1 |
| Sreedhara et al. (2022) | 1 | × | × | 2 | × | 1 | × | 0 | 1 |
| Vanhatalo et al. (2007) | 1 | 2 | 0 | × | 2 | 1 | 2 | 2 | 1 |
| Wilkins et al. (2025) | 2 | 0 | 0 | × | 0 | 1 | 2 | 2 | 2 |
| Wright et al. (2017) | 2 | 2 | 0 | 2 | 2 | 1 | 2 | 0 | 1 |
| Wright et al. (2019) | 2 | 2 | 0 | × | 2 | 1 | 2 | 0 | 1 |
| Zagatto et al. (2019)  B = both; V = validity; R = reliability; 1 = yes; 0 = no; 2 = no agreement; × = not appropriate for study; Items: Item 1 = description of sample; Item 3 = explanation of reference standard; Item 7 = short time period between tests; Item 8 = stability of variable being between repeated measures; Item 9 = reference standard independent of index test; Item 10 = test execution described in detail; Item 11 = reference standard described in detail; Item 12 = withdrawals explained; Item 13 = statistical methods appropriate for purpose | 1 | 2 | 0 | × | 2 | 1 | 2 | 0 | 1 |
