# Supplementary material for: The 3-min all-out test in cycling: a systematic review and meta-analysis on its reliability and validity
Source: Eur J Appl Physiol. 2026 Jul 21;126(9):4673–91. doi: 10.1007/s00421-026-06356-w (PMC13541879; doi:10.1007/s00421-026-06356-w)
Supplement: Supplementary file 3 [file 421_2026_6356_MOESM5_ESM.docx]

**Appendix 5 – Additional information about methodological characteristics for reliability outcomes**

| Study | 3-min  all-outs | age  (in years) | V̇O_2_ | Famila-rization | warm-up | Before test start | Resistance | Cadence |
| --- | --- | --- | --- | --- | --- | --- | --- | --- |
| Karsten et al. (2013) | 7 visits 3 tests | 33 ± 7 | no | yes | 5 min at 100 W + 5 min passive rest + 3 min of unloaded cycling | 3 min of unloaded cycling | Changing  (isokinetic) | Preferred |
| Burnley et al. (2006) | 6 visits 2 tests | 27 ± 7 | yes | Yes | 5 min at 100 W + 5 min passive rest + 3 min of unloaded cycling | 3 min of unloaded cycling | 50% ΔGET and V̇O2max/preferred cadence² | Preferred |
| Clark et al. (2018) | 5 visits  3 tests | 31 ± 6 | no | yes | 3 min baseline at 30 W before the 2 h intensity | 2 h bout with 5 s reduced to 20 W | 50% ΔGET and V̇O2peak/preferred cadence² | 70-90 rpm |
| De Lucas et al. (2014) | 5 visits 4 tests | 27.1 ± 4.5 | yes | partly | 5 min at GET + 5 min complete rest + 3 min unloaded cycling | 3 min of unloaded cycling | Changing  (isokinetic) | 60 rpm 100 rpm |
| Johnson et al. (2011) | 3 visits 2 tests | 22 ± 2 | yes | no | n.r. | 50 Watts 30 s before | 50% ΔGET and V̇O2peak /preferred cadence² | Preferred |
| Maunder et al. (2022) | 3 visits 2 tests | 39 ± 9 | no | yes | 10 min at 100W | n.r. | Changing (freely geared) | Changing (freely geared) |
| Sreedhara et al. (2023) | 5 visits 4 tests | 42 ± 10 | no | yes | 5 min at 100 W at 80 rpm + 5 minute passive rest + 3 min unloaded pedaling at 80 rpm | 3 min unloaded pedaling at 80 rpm | n.r. (shifting gears prohibited) | n.r. |
| Wright et al. (2017) | 8 visits 4 tests | 32 ± 6.6 | yes | yes | 10 min at 100W | 30 s of unloaded cycling | 50% ΔGET and V̇O2peak/preferred cadence² | Preferred |

Abbreviations: GET = gas exchange threshold; n.r. = not reported; rpm = revolutions per minute; V̇O_2peak_ = peak oxygen uptake; V̇O_2max_ = maximal oxygen uptake
