# Supplementary material for: The 3-min all-out test in cycling: a systematic review and meta-analysis on its reliability and validity
Source: Eur J Appl Physiol. 2026 Jul 21;126(9):4673–91. doi: 10.1007/s00421-026-06356-w (PMC13541879; doi:10.1007/s00421-026-06356-w)
Supplement: Supplementary file 4 [file 421_2026_6356_MOESM6_ESM.docx]

**Appendix 6 – Additional information about methodological characteristics for validity outcomes**

| Study | age  (in years) | warm-u | V̇O_2_ | Familari-zation | Resistance | Cadence | Before test start |
| --- | --- | --- | --- | --- | --- | --- | --- |
| Bartram et al. (2017) | 20 ± 1 | n.r. | no | no | Changing (freely geared) | Changing (freely geared) | n.r. |
| Karsten et al. (2013) | 33 ± 7 | 5 min at 100 W  + 5 min passive rest + 3 min  of unloaded cycling | no | yes | Changing  (isokinetic) | Preferred | 3 min of unloaded cycling |
| Black et al. (2014) | 33.8 ± 8.2 | n.r. | no | yes | 50% ΔGET and V̇O_2peak_/preferred cadence² | Preferred | 4 min of unloaded pedalling |
| Dekerle et al.(2014) | 21 ± 2 | 5 min at 80% LT | no | yes | Changing  (isokinetic) | 60 rpm 100 rpm | 1 min of unloaded baseline pedalling |
| McClave et al. (2011) | 40.5 ± 10.7 | 10 min warm-up | yes | no | power output identified at the midpoint between VEbp and V̇O2max | Preferred | No rest |
| Vanhatalo et al. (2007) | 33 ± 9 | 5 min at 100 W + 5 min rest | yes | yes | 50% ΔGET and V̇O_2peak_/preferred cadence² | Preferred | 3 min of unloaded baseline |
| Wright et al. (2019) | 30 ± 5 | 10 min  at 100 W | yes | Completed 4 tests  within the last 12 months | 50% ΔGET and V̇O_2max_/preferred cadence² | Self-selected | 30 s of unloaded cycling |
| Barker et al. (2011) | 14.5 ± 0.3 | 5 min at 80% GET + 3x10-s sprints + 20 min seated rest + 6 min at 10 W | yes | partly | 50% between GET and V̇O_2peak_ | 75 rpm | 6 min at 10 W baseline |
| Clark et al. (2016) | 26 ± 9 | yes but n.r. | Yes | No | Changing (freely geared) | Preferred | n.r. |
| Clark et al. (2019) | 31 ± 10 | 2 h preconditioning | no | yes | 50% ΔGET and V̇O_2max_/preferred cadence² | Preferred | 3 min of unloaded baseline |
| Goulding et al.(2021) | 28 ± 8 | 3 min at 30 W Ramp test with linear increase of 30 W/min | yes | no | work-rate = linear factor × cadence² | n.r. | Passive rest for time to switch mode |
| Maunder et al. (2022) | 39 ± 9 | 10 min at 100W | no | yes | Changing (freely geared) | Changing (freely geared) | n.r. |
| Wilkins et al. (2025) | 21 ± 1 | 5 min of self selected warm up | no | no | Changing (freely geared) | Changing (freely geared) | no rest |
| Wright et al. (2017) | 32 ± 6.6 | 10 min at 100W | yes | yes | 50% ΔGET and V̇O_2max_/preferred cadence² | Preferred | 30 s of unloaded cycling |
| Zagatto et al. (2019) | 28.1 ± 4 | 10 min of 30-70% of VO2max and 10 min rest afterwards | Yes | Yes | 50% ΔGET and V̇O_2max_/preferred cadence² | Preferred | 10 min rest |

n.r. = not reported; LT = lactate threshold; GET = gas exchange threshold; V̇O_2peak_ = peak oxygen uptake; V̇O_2max_ = maximal oxygen uptake; rpm = revolutions per minute
